# Atlanta Medical College

**Published:** 1860-06

**Authors:** 


### EDITORIAL AND MISCELLANEOUS.

### Atlanta Medical College.

The Sixth Session in the Atlanta Medical College, commenced with an Introductory Lecture from Dr. J. G. Westmoreland, on the first Monday in May. This Address will be found in the present number of the Journal, and will be found highly instructive and interesting. Dr. Westmoreland admits the defects in the present plan of Medical education, and shows conclusively that there is no remedy but more rigid exactions in the examinations for graduation. Indeed, the practical illustration and demonstration is presented in graduates of the University of Virginia, who are known to be *the most thoroughly educated Medical men in the United States*, and, yet, are permitted to apply for a degree at the end of a single term of nine months, without reference to the period of study or of attendance upon Lectures.

We have repeatedly contended for *the separation of the teaching and licensing power in Medical education*, and honestly believe that there is no other mode under heaven, that holds out any prospect of improvement in this matter, in Institutions dependent upon the patronage of Students for support. It is true that the Atlanta Medical College has done something for Medical education in taking the initiative in the adoption of a systematic plan of *Examination*, in some degree similar to the course pursued at the University of Virginia, and is sincerely desirous to elevate the standard of requisitions for graduation, but finds herself constantly embarrassed by the almost universal rule of the Medical Colleges of the land, to allow students to receive diplomas at the end of a second term of Lectures, without regard to qualifications. For ourselves, however, we are determined that no responsibility shall attach for having aided in conferring the degree upon those who are, in our judgment, unworthy to occupy a place in the ranks of the profession. We commend Prof. Westmoreland's Address to the attention of our readers.

The class in attendance upon the course of Lectures is already a large one, (representing ten States) and is, in the highest degree, creditable in appearance and conduct.

